# Supplementary material for: Antibody Responses to SARS-CoV-2 and Common HCoVs in Hemodialysis Patients and Transplant Recipients: Data from the Dominican Republic
Source: Vaccines (Basel). 2025 Sep 11;13(9):965. doi: 10.3390/vaccines13090965 (PMC12474128; doi:10.3390/vaccines13090965)
Supplement: Supplementary file 1 [file vaccines-13-00965-s001.zip › vaccines-3790404-supplementary.pdf]

Supplementary Material

IMMEDIATE

Antibody Responses to SARS-CoV-2 and Common HCoVs in Hemodialysis Patients and Transplant Recipients: Data from the Dominican Republic.

Lisette Alcantara Sanchez[1], Eloy Alvarez Guerra[2], Dongmei Li[1], Samantha M. King[3], Shannon P. Hilchey[3], Qian Zhou[3], Stephen Dewhurst[4], Kevin Fiscella[5,‡], and Martin S. Zand[1,3,6,‡,*]

[1] Clinical and Translational Science Institute, University of Rochester, Rochester NY
[2] Instituto Nacional de Coordinación de Trasplante, Santo Domingo, Dominican Republic
[3] Department of Medicine, Division of Nephrology, University of Rochester, Rochester NY
[4] Department Microbiology and Immunology, University of Rochester, Rochester NY
[5] Department of Family Medicine, University of Rochester, Rochester NY
[6] Department of Public Health Sciences, University of Rochester, Rochester NY

Characteristic	Hemodialysis, N = 13[1]	Transplant, N = 24[1]	p-value[2]
**Age**			0.312
18-24	1 (7.7%)	3 (12.5%)	
25-44	5 (38.5%)	11 (45.8%)	
45-65	7 (53.8%)	10 (41.7%)	
**Sex**			0.225
Female	4 (30.8%)	10 (41.7%)	
Male	9 (69.2%)	14 (58.3%)	
**Vaccine type (Prime schedule)**			0.03
BNT162b2	7 (63.6%)	11 (45.8%)	
CoronaVac	4 (36.4%)	13 (54.2%)	
Not Reported	-	-	
**Booster schedule**			0.767
Heterologous	1 (50.0%)	6 (54.5%)	
Homologous	1 (50.0%)	5 (45.5%)	
Not Reported	-	-	
**Covid positive before first fingerstick**			0.314
Yes	6 (46.2%)	15 (62.5%)	
No	7 (53.8%)	9 (37.5%)	
**Covid positive during the study**			0.523
Yes	2 (15.4%)	5 (20.8%)	
Not Reported	11 (84.6%)	19 (79.2%)	
**Hospitalization**			0.031
Yes	3 (23.1%)	1 (4.2%)	
No	-	-	
**Cause of ESRD**			<0.001
Hypertension (HTN) w/o other	5 (38.5%)	8 (33.3%)	
HTN and Pregnancy	2 (15.4%)	2 (8.3%)	
Diabetes Mellitus (DM)	2 (15.4%)	2 (8.3%)	
Congenital Anomalies	-	3 (12.5%)	
Rheumatic Heart Disease	-	2 (8.3%)	
Glomerulopathy	1 (7.7%)	5 (20.8%)	
Lupus	-	1 (4.2%)	
Others	3 (23.1%)	1 (4.2%)	
**Flu vaccine after dialysis or transplant**			<0.001
Yes	4 (30.8%)	21 (87.5%)	
No	-	-	
**Hepatitis B vaccine**			<0.001
Yes	7 (53.8%)	24 (100.0%)	
No	-	-	

[1] n (%)

[2] Fisher's exact test; Pearson's Chi-squared test

Table S1. End-stage kidney disease and transplant cohort characteristics. Reproduced from [23]. This table summarizes clinical characteristic comparisons between hemodialysis patients (HD, N = 13) and transplant recipients (TR, N = 24). No significant differences were observed in age distribution or sex ($p = 0.312$ and $p = 0.225$, respectively). In contrast, significant differences were found in vaccine type ($p = 0.03$), hospitalization rates ($p = 0.031$), cause of end-stage renal disease (ESRD, $p < 0.001$), influenza vaccination after dialysis or transplant ($p < 0.001$), and Hepatitis B vaccination ($p < 0.001$). Specifically, HD patients more frequently received the BNT162b2 vaccine, whereas TR patients predominantly received CoronaVac. Hospitalizations were more common in the HD group. The underlying cause of ESRD differed notably, with TR patients showing higher rates of congenital anomalies and glomerulopathy. Influenza and Hepatitis B vaccination rates were also significantly higher among TR patients.

Characteristic	Hemodialysis, N = 13
**Hemodialysis access**	
AV Fistula	4 (30.8%)
Catheter	3 (23.1%)
Catheter & AV Fistula	4 (30.8%)
Not reported	2 (15.4%)
**Dialysis vintage, mo**	
<12 months	1 (7.7%)
13- 24 months	7 (53.8%)
25 - 48 months	3 (23.1%)
>49 months	1 (7.7%)
Not reported	1 (7.7%)

Table S2. Hemodialysis characteristics. Reproduced from [23]. This table summarizes the hemodialysis characteristics of 13 subjects, focusing on access type and treatment duration. Among them, 4 subjects (30.8%) used an AV fistula, 3 subjects (23.1%) used a catheter, and 4 subjects (30.8%) used both types of access. Most subjects (53.8%) had been on dialysis for 1-2 years, with the remaining subjects having either shorter or longer duration. Overall, the AV fistula was the most commonly used access method.

Characteristic	Transplant, N = 24
**Transplant time**	
<6 months	2 (8.30%)
7-12 months	3 (12.50%)
13 - 24 months	3 (12.50%)
>25 months	16 (66.70%)
**Induction therapy <12 Months**	
Basiliximab	4 (16.70%)
Thymoglobulin	2 (8.30%)
**Immunosupression (current)**	
Certican (Everolimus)	17 (70.80%)
Cyclosporine	19 (79.20%)
Mycophenolate Mofetil	9 (37.50%)
Prednisone	6 (25.00%)
Tacrolimus	9 (37.50%)
**Type of organ donation**	
Living donation	16 (66.70%)
Deceased donation	8 (33.30%)
**Organ rejection (Transplant 2020 - 2021)**	
Acute graft	2 (8.30%)
Chronic graft	1 (4.20%)
**Rejection treatment**	
Rituximab	2 (8.30%)
Tacrolimus	3 (12.50%)
Not reported	2 (8.30%)

Table S3. Transplant Characteristics. Reproduced from [23]. The table summarizes transplant characteristics for 24 subjects. Most had their transplant over 3 years ago (66%). The most common immunosuppressants were Cyclosporine (79.2%) and Certican (70.8%). Living donations made up 66.7% of transplants. In 2020-2021, 8.3% experienced acute rejection and 4.2% had chronic rejection, with Tacrolimus (12.5%) and Rituximab (8.3%) used for rejection treatment.

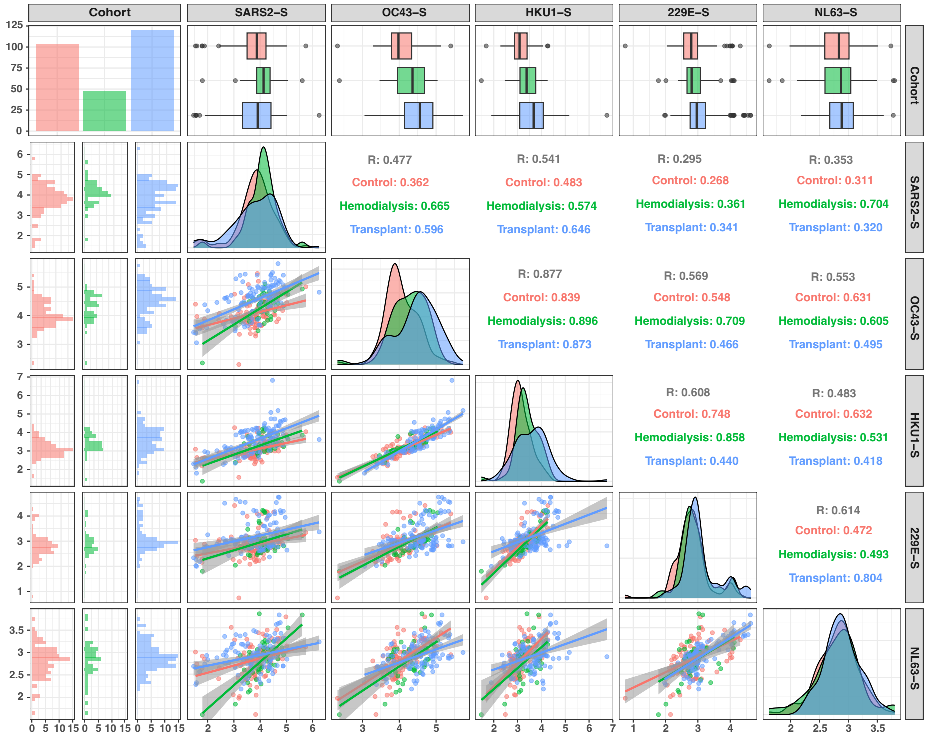

Figure. S1. Correlations of Anti-Spike IgG responses across SARS-CoV-2 and common coronaviruses (HCoVs) in control (n = 33), hemodialysis (n = 13), and transplant recipient (n = 24) cohorts. HD patients exhibit the strongest and most consistent correlations, particularly between OC43-S and HKU1-S ($R = 0.896$) and between SARS-CoV-2 and OC43-S ($R = 0.665$), indicating enhanced cross-reactivity. TR patients show overall weaker correlations, though strong associations are observed between OC43-S and HKU1-S ($R = 0.873$) and between SARS-CoV-2 and HKU1-S ($R = 0.646$). The CO group demonstrates moderate correlations across most comparisons. Density and scatter plots illustrate broader and elevated anti-S IgG responses in HD compared to CO and TR, consistent with heightened humoral reactivity in HD. All correlation coefficients (R) reflect Pearson correlations. Antibody concentrations are shown on a log scale.

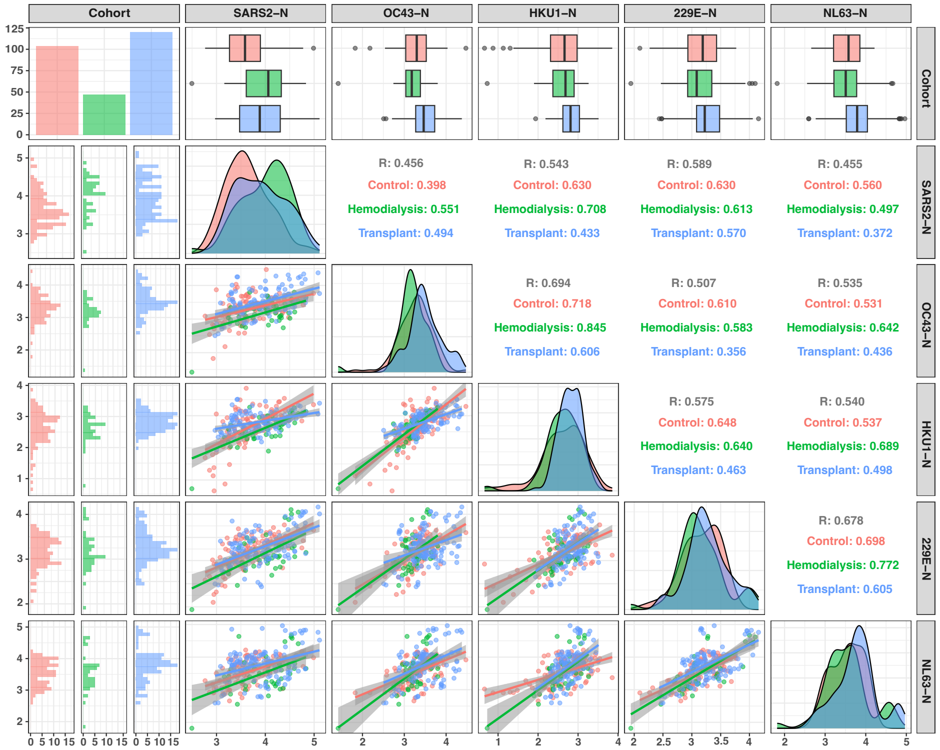

Figure. S2. Correlations of Anti-Nucleocapsid IgG responses across SARS-CoV-2 and common coronaviruses (HCoVs) in control ($n = 33$), hemodialysis ($n = 13$), and transplant recipient ($n = 24$) cohorts. HD patients exhibit the strongest and most consistent anti-N IgG correlations across all coronaviruses, including SARS-CoV-2, suggesting enhanced cross-reactivity or immune activation. Within the HD group, SARS-CoV-2 anti-N IgG shows its highest correlations with HKU1 ($R = 0.708$) and OC43 ($R = 0.551$). In contrast, CO subjects display moderate correlations, while TR recipients show the weakest and most variable responses, likely due to immunosuppression. Scatter and density plots support these observations, revealing broader and elevated anti-N IgG levels in HD, particularly for SARS-CoV-2. Additionally, strong correlations are observed in HD between OC43 and HKU1 ($R = 0.845$) and between HKU1 and 229E ($R = 0.772$), indicating robust cross-reactivity or shared immune imprinting. All correlation coefficients (R) reflect Pearson correlations. Antibody concentrations are shown on a logarithmic scale (left).

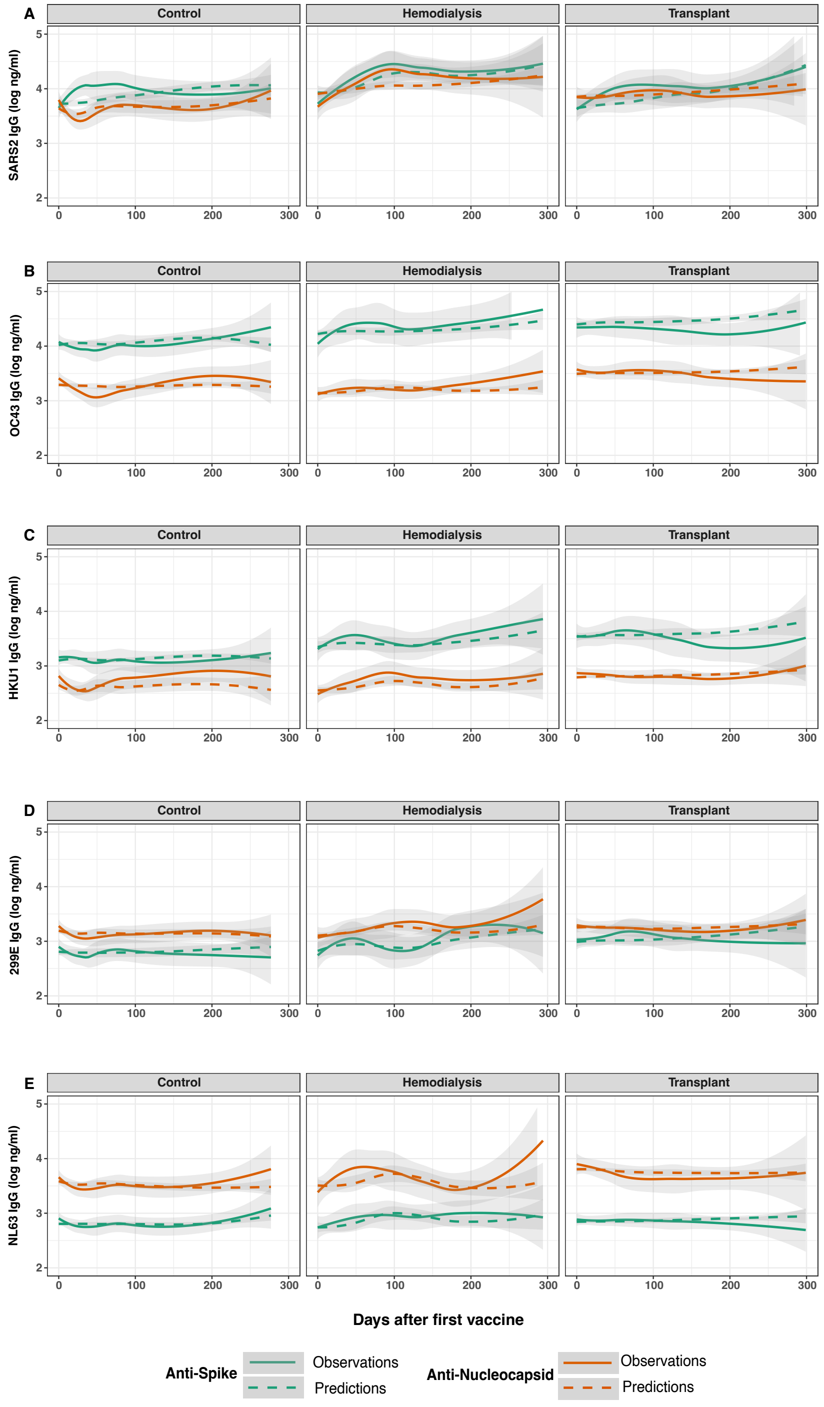

Figure. S3. MLMM Performance: Anti-Spike and Anti-Nucleocapsid for SARS-CoV-2 and HCoVs. Antibody Response Predictions vs. Observations. Total anti-S (green lines) and anti-N IgG (orange lines) distribution measured days after first vaccine dose by multiplex assay. Observation (solid lines), predictions (dashed lines) Data were fitted to a spline curve for each group, with 95% confidence levels noted by the gray bands. Day 0 corresponds to the day of the first vaccine dose, mean time for second (36.6 ± 34.1 days) and booster vaccinations (154.3 ± 41.1 days).
